# PPAR-Gamma Agonist Pioglitazone Reduced CD68+ but Not CD163+ Macrophage Dermal Infiltration in Obese Psoriatic Patients

**DOI:** 10.1155/2020/4548012

**Published:** 2020-05-01

**Authors:** Ya. O. Yemchenko, V. I. Shynkevych, K. Ye Ishcheikin, I. P. Kaidashev

**Affiliations:** Ukrainian Medical Stomatological Academy, Poltava 36024, Ukraine

## Abstract

**Background:**

Macrophages are of great importance in the development of obesity and psoriasis. Signaling via PPAR-*γ* in certain macrophage populations is associated with M2-like features and anti-inflammatory profile. In this research, we evaluated the anti-inflammatory action of pioglitazone by the immunohistochemical study of M1 and M2 macrophages in psoriasis-affected skin in obese patients.

**Methods:**

We used immunohistochemistry to characterize CD68+ and CD163+ macrophages and pathomorphological description of skin biopsy, obtained from 6 obese psoriatic patients before and after treatment with 15, 30, and 45 mg pioglitazone, once a day during 6 months. Two patients with conventional therapy and without pioglitazone served as control.

**Results:**

Generally, CD163+ cell quantities in psoriasis-affected skin significantly dominated over CD68+ before and after all treatment regiments. Among patients who received pioglitazone, some of them clearly responded to treatment from lowest to highest doses by decreasing CD68+ cells. In the group with 30 mg pioglitazone regiment, we detected a significant reduction of CD68+ cells in dermal infiltrates: CI 95% (16–32) before versus CI 95% (2–7) after treatment. Pioglitazone dose escalation led to certain normalization of skin morphology.

**Conclusion:**

The immunohistochemical study allows us to show the anti-inflammatory effect of pioglitazone in psoriatic obese patients, which can be mediated by reducing the number of СD68+ macrophages, but not СD163+ macrophages, in the affected dermis.

## 1. Introduction

Psoriasis is a life-long, incurable, disfiguring, disabling, stigmatizing disease with a worldwide prevalence of about 2% [1]. It can manifest in many different forms and often accompanied by obesity [2].

The etiology and pathogenesis of psoriasis include many immunological mechanisms [3, 4], in which macrophages (M*φ*s) and their different populations are involved in the inflammatory cascade [5]. Сomorbid obesity further complicates immunological disorders in psoriasis [2, 6].

The clinical studies showed the therapeutic effects of PPAR-*γ*-agonist pioglitazone (PGZ) in the treatment of cutaneous and metabolic pathologies in psoriasis [7, 8], but mechanisms of such effects remain unclear. PGZ is a specific agonist of PPAR-*γ* from the thiazolidinedione group, in which PPAR-*γ* stimulation led to a reduction in the expression of NF*κ*B in mononuclear cells [9]. Macrophages PPAR-*γ* can be one of the possible targets for PGZ [10].

In addition, M*φ*s are of great importance in the development of obesity and psoriasis. Accumulated researches showed that pathogenesis of obesity and psoriasis has a common trait that causes a systemic inflammatory response. Excessive accumulation of adipose tissue, which produces adipokines and cytokines, is a source of chronic inflammation due to the involvement of macrophages capable of secreting and activating cytokines for inflammation. It is proved that fatty tissue has almost all known Toll-like receptors (TLRs). TLR ligands are both LPS of microorganisms and saturated fatty acids. Activation of fatty tissue TLRs leads to increased synthesis of adipokines, cytokines, and chemokines and stimulates the subsequent expression of TLRs [11]. Molecular mechanisms of chronic systemic inflammation are associated with proinflammatory nuclear transcription factor NF*κ*B [12] and anti-inflammatory activity of PPARs, which directly modulate the activity of genes responsible for the condition and function of adipose tissue, lipid metabolism, the activity of inflammatory cells, and their production of cytokines, adhesion factors, cell differentiation, and apoptosis. PPAR-activated receptors are central between lipids and inflammation because lipids that stimulate chronic inflammation are PPAR-activating ligands also [13].

There are two main clinically significant subpopulations of M*φ*s: classically (M1) and alternatively activated (M2) [10, 14]. M1 and M2 M*φ*s have the ability to exhibit the polar-opposite M2/heal and M1/kill functions [15]. M*φ*s polarization occurs against the background of significant metabolic changes [10]. In different diseases, the M1/M2 ratio can change, as does the expression profile of these cells, which has a pathogenic role [16, 17].

PPAR-*γ* tickling of these subpopulations may lead to the development of anti-inflammatory effects [18–20].

CD68 and CD163 are used to identify M*φ*s in tissue sections [21]. CD163 is a hemoglobin-haptoglobin complex-binding scavenger receptor and one of several macrophage markers classified as markers of alternatively activated M*φ*s [22]. CD68 molecule is functionally important for M1 macrophages. As a receptor for oxidized low-density lipoproteins, it can activate M1 phagocytosis and promote proinflammatory cytokine production. CD68 is highly expressed on human monocytes and tissue M*φ*s [21, 23]. The previous study demonstrated that macrophage marker CD68 was increased in psoriasis compared to normal skin, and coexpressed with CD163 [24].

Thus, in this research, we evaluated the anti-inflammatory action of pioglitazone by the immunohistochemical study of M1 and M2 M*φ*s in psoriasis-affected skin.

## 2. Materials and Methods

### 2.1. Subject Recruitment

Prior to the study, the approval from the Academy's Bioethics Commission was obtained.

The study included 8 patients with the verified diagnosis of extensive psoriasis vulgaris, advanced stage, and moderate severity with concomitant obesity 30 ≤ BMI < 40 kg/1.73 m^3^ (3 women and 5 men, aged from 40 to 60, the average age of 52 years). All patients signed a voluntary informed consent to participate in the study.

Patients were stratified and randomized 1 : 1 : 1 : 1; each group included 2 patients. The 1st group of patients received standardized conventional therapy—sedation, detoxifying agents, antihistamine, hepatoprotectors, vitamins, and 1-2% salicylic ointment 2 times a day topically. Patients of groups 2, 3, and 4 were prescribed pioglitazone at a dose of 15, 30, and 45 mg, respectively, orally once a day for 28 days, additionally to the treatment protocol.

### 2.2. Punch Biopsy

We conducted an in-depth descriptive study of macrophage subpopulations on skin biopsies. The samples were obtained by punch biopsy of psoriatic plaques from the skin areas of lower extremities exactly before treatment and on the 28th day. Punch biopsy was performed using a special tubular scalpel (Dermo-punch) with a diameter of 3 mm, under local infiltration anesthesia with lidocaine hydrochloride 2%. The biopsies were fixed in a 10% solution of neutral buffered formalin and embedded in paraffin.

### 2.3. Immunohistochemical Studies

We performed immunohistochemical studies using the streptavidin peroxidase method. Paraffin sections, 3 *μ*m thick, were deparaffinized and dehydrated, antigens were recovered in citrate buffer in the microwave oven, and endogenous peroxidase was blocked. Further, the sections were incubated at 4°C overnight with murine monoclonal antibodies anti-CD68 (1 : 30, clone PG-М1, REF PD M065-S, Diagnostic BioSystems, USA) and anti-CD163 (1 : 100, clone 10D6, REF Mob460-01, Diagnostic BioSystems, USA). Afterwards, the sections were treated in two steps with the Mouse/Rabbit PolyVue™ HRP/DAB Detection System (Diagnostic BioSystems, USA), with visualization by chromogen; the nuclei were counterstained with Mayer's haemalaun and enclosed under the cover glass. We used Antibody Diluent buffer as a negative control instead of primary antibodies, and lymph node tissues were used as a positive control.

Quantitative indicators were obtained by counting immunopositive CD68+ and CD163+ cells over the entire field of view with a large magnification lens ×40 (high power field, HPF) of the dermal region. We took into account all obtained quantitative individual data from all fields of view without calculating the mean. Microphotographs were obtained using the ZEISS Axio Lab.A1 microscope, Carl Zeiss MicroImaging GmbH (mg; ×200, ×400).

### 2.4. Statistical Analysis

We conducted that the statistical analysis of comparisons between groups was performed using the GraphPad Prism 5 software with parametric *T*-test (if normal distribution) and nonparametric methods: Wilcoxon rank data analysis for dependent variables and Spearman correlation.

## 3. Results

**Figure 1:**
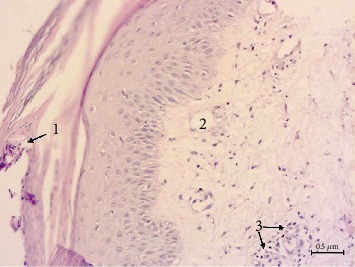
Pathomorphology of psoriasis-affected skin: Munro's microabscesses (1), dilated capillaries in the dermal papillae (2), and lymphohistiocytic infiltrate (3). Hematoxylin-eosin staining, mg, ×200. Scale bar, 0.5 *μ*m.

The pathomorphological pattern of the psoriasis-affected specimens in all patients had characteristic signs of this disease. We detected the typical changes of the epidermis, such as accumulation of neutrophils in the stratum corneum of the epidermis: Munro's microabscesses, decreased thickness of the epidermis as compared to the length of dermal papillae, regular acanthosis, often with dentate ridges of the epidermis, and dilated capillaries in the papillae. Lymphohistiocytic infiltrates of various densities were localized subepidermally in the dermal papillae (Figure 1).

**Figure 2:**
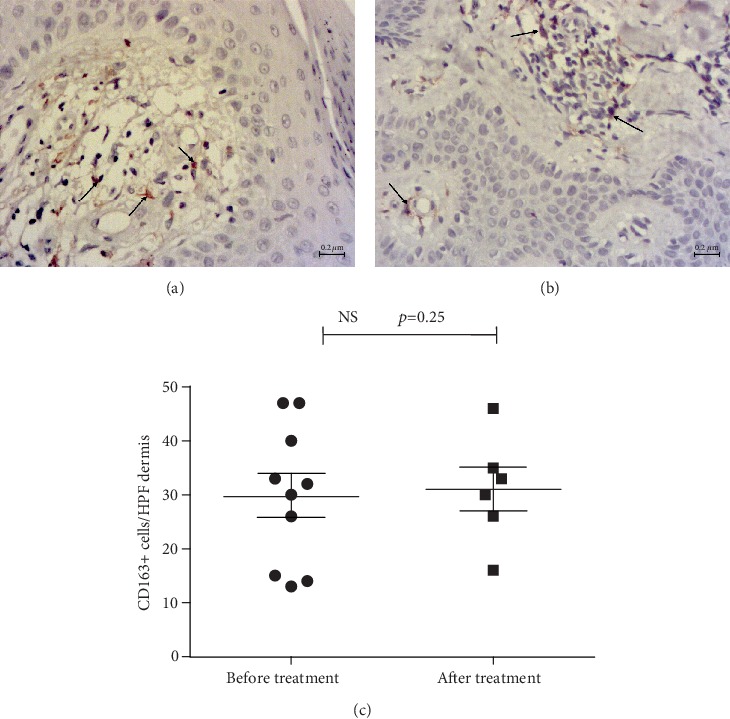
Immunohistochemical detection of CD163+ cells in the psoriatic plaque biopsy specimen before (a) and after treatment with 30 mg pioglitazone (b). CD163+ cells (arrows) are rather tightly represented in the dermal papillae surrounding the vessels. Contrasting: Mayer's haemalaun, mg, ×400. Scale bars, 0.2 *μ*m. CD163+ сell number in all groups did not differ significantly before and after treatment (c).

**Figure 3:**
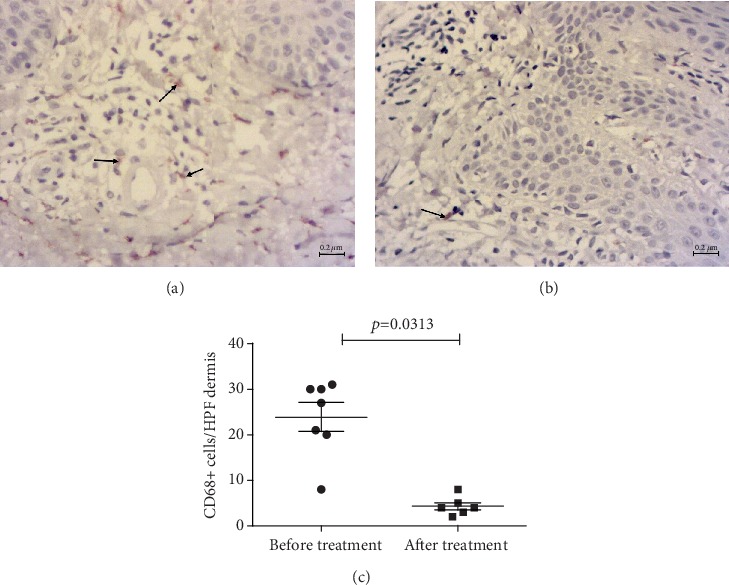
Immunohistochemical detection of CD68+ cells in psoriatic plaque biopsy specimens. (a) CD68+ cells (arrows) in the infiltrate and the reticular layer of the dermis before treatment. (b) Single CD68+ cells (arrow) after the treatment with 30 mg pioglitazone. Contrasting: Mayer's haemalaun, mg, ×400. Scale bars, 0.2 *μ*m. (c) CD68+ cells reduced significantly (Wilcoxon method).

**Figure 4:**
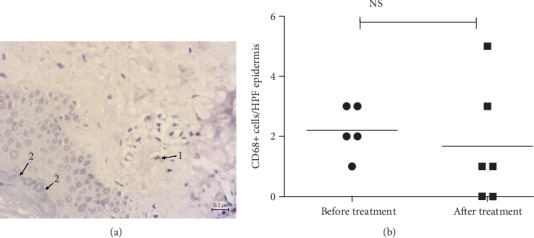
Immunohistochemical detection of CD68+ cells in skin biopsy specimens after treatment of psoriasis with pioglitazone 45 mg. (a) Single immunopositive cell (1) and granular layer (2). Contrasting: Mayer's haemalaun, mg, ×400. Scale bar, 0.2 *μ*m. (b) CD68+ сell number in the patient did not differ significantly.

In psoriatic skin lesions, general characteristics of CD68+ and CD163+ were as follows: immunoreactivity was observed in the form of brown granular staining of cells, which revealed their different size and shape features; CD68+ and CD163+ cells in psoriatic skin were localized in the dermal papillae and deeper dermis, along and around the dilated superficial vessels, as well as in the composition of lymphohistiocytic infiltrates. We found the largest number of immunopositive cells in the papillary and reticular layers of the dermis (Figures 2–4).

Visually, the number of CD163+ cells dominated in all samples. The assessment of CD68+/CD163+ ratio, obtained by the *T*-test for dependent variables, confirmed a significantly higher fraction of CD163+ cells (*p* = 0.0002), with no significant correlation between CD68+ and CD163+ quantities. The average number of CD68+ was 10 cells (per HPF of papillary and reticular layers of dermis), confidential interval (CI) 95% (6–14), whereas the number of CD163+ was 22 cells, CI 95% (17–26).

After treatment, the ratio did not change, and CD163+ M*φ*s prevailed over CD68+ (*p* = 0.0002, Wilcoxon method) in all groups.

Numerous CD163+ cells in psoriasis were observed in at least two forms: small-sized cells with a small nucleus, as in lymphocytes, and relatively large branched cells; these varieties persisted after treatment. Both types of cells were localized in essentially the same areas and had different shapes depending on the position. Hence, the slightly larger ones were on the tips of the papillae, whereas smaller cells were in the composition of lymphohistiocytic infiltrates, and/or flattened spindle-shaped—in the reticular layer. The typical localization was along the vessels of the dermal papillae, which were observed specifically for CD163+ cells, where they formed a kind of the first line (Figure 2), as compared to CD68+ (Figure 3). CD68+ M*φ*s also occurred in different shapes and sizes, slightly sparse in infiltrates, and presented in the dermal papillae, sometimes not in all, around the capillaries (Figure 4).

The morphological and quantitative comparison of CD163+ cells before and after treatment demonstrated no significant differences in each group and in total (Figure 2).

In the group with conventional therapy, comparison of CD68+ cells before and after treatment demonstrated no statistically significant changes or morphologic differences (*p* = 0.343, data not shown).

A personalized approach justifies close attention to each patient. Therefore, the study revealed the patient who clearly responded to the treatment with the 15 mg of PGZ, in whom CD68+ cells almost completely disappeared after treatment.

In the group with 30 mg PGZ, the statistical comparison of CD68+ cells (Wilcoxon method) showed a significant decrease. Treatment with 30 mg PGZ use decreased the number of CD68+ cells from CI 95% (16–32) to CI 95% (2–7) significantly (Figure 3).

Besides, in one patient after the treatment with 45 mg of PGZ, dermal papillae presented on the preparation were smoothed, epidermal ridges were rounded, and orthokeratosis (morphological signs of some normalization of the skin condition) and only single CD68+ cells were observed, which can be interpreted as a decrease in the infiltration density (Figure 4).

## 4. Discussion

The involvement of M*φ*s in the pathogenesis of psoriasis is a long-standing but actual research issue. In psoriasis, M*φ*s are involved in the perception of danger signals. Their activation by the proinflammatory cytokines IL-6 and TNF-*α* and chemokines CXCL8, CCL5, CXCL1, and CXCL2 involves other inflammatory cells [25]. It is known that the number of M*φ*s increases significantly in psoriasis-affected tissues [26]. The M1/M2-type macrophages drove T cell differentiation to Th1- or Th2-like phenotype, respectively. Such macrophage-innate activities are the central directing element in immune responses which is a drastic change in understanding how immune systems operate. Most importantly, this revelation is opening up whole new approaches to immunotherapy [27].

The data on M*φ*s polarization under the influence of PPAR-*γ* agonists and antagonists are steadily growing, mainly in animal models [18-20]. Signaling via PPAR-*γ* in the individual M*φ*s populations is associated with M2-like features, including the anti-inflammatory ones [28].

Thus, we investigated the influence of PPAR-*γ* agonist PGZ on M1 and M2 M*φ*s of psoriasis-affected skin, by immunohistochemical study. Our data showed a decrease in the CD68+ M*φ*s population by different doses of PGZ. CD68 marker was used in a recent study as one of the identifiers for inflammatory M1 [29]. The reduction of CD68+ M*φ*s has correlated with clinical success in psoriatic patients. The numbers of phosphorylated I*κ*B*α* activated CD68+ M*φ*s were reduced in psoriasis plaques after treatment with new biomaterials based on nanoparticles significantly [29]. Therefore, a decrease of CD68+ M1 can explain the success of PGZ treatment.

In obese psoriatic patients, CD163+ cells were the predominant population in the affected skin, compared to CD68+. Conventional treatment did not influence this ratio. The prevalence of CD163+ cells as alternative activated M*φ*s goes in parallel with angiogenesis de novo in psoriatic skin. The descriptive morphological comparison of CD163+ cells before and after the treatment with PGZ showed no drastic changes.

Our data support the idea that CD163+ cells drive immune-mediated proliferation in these patients [30]. On the other hand, the controversial data about CD163+ cell classical activation in psoriasis were published [24].

PPAR-*γ*-dependent activation of macrophages is obviously capable of restoring the vital cleaning or clearance functions of macrophages in some infections, preventing excessive inflammation in septic conditions [28]. Therefore, the administration of PGZ may also influence CD163+ M2 M*φ*s, although their quantitative and morphological changes could not be recorded in this study.

Additional treatment with PGZ reduced the number of CD68+ cells starting at a dose of 15 mg; statistical significance was registered at a dose of 30 mg. Furthermore, in the dose of 45 mg, there were also signs of the decreased density of CD68+, together with morphological manifestations of regression of psoriatic plaques: thickening of the epidermis, orthokeratosis, reduced height of the dermal papillae, and lack of branching of the epidermal ridges. Thus, PGZ dose escalation led to certain normalization of skin morphology.

CD68 is a type I integral membrane protein with a heavily glycosylated extracellular domain and binds to tissue- and organ-specific lectins or selectins. The protein is a member of the scavenger receptor family, with typical functions which are cellular debris removal, phagocytosis promotion, and M*φ*s recruitment. Thus, СD68 contributes to inflammation support [31].

Taken together with clinical indicators in the dynamics of psoriasis treatment with PGZ, such as a decrease of PASI index and reduction of relapse rate up to 1-2 times per year [7, 8, 32], decreasing of CD68+ cells indicates inhibition of inflammation locally.

The limitations of the study were a small number of patients due to ethical reasons and using of M*φ*s markers that can partially overlap in pathology [16, 17, 24, 29].

Further research will be targeted on the investigation of M1/M2 cytokine levels under PGZ influence on obese psoriatic patients.

## 5. Conclusions

The immunohistochemical study allows us to show the anti-inflammatory effect of pioglitazone in psoriatic obese patients, which can be mediated by reducing the number of СD68+ macrophages, but not СD163+ macrophages, in the affected dermis.

## Data Availability

The data used to support the findings of this study are available from the corresponding author upon request.
